# Disturbance Robust Attitude Stabilization of Multirotors with Control Moment Gyros

**DOI:** 10.3390/s24248212

**Published:** 2024-12-23

**Authors:** Youyoung Yang, Sungsu Kim, Kwanghyun Lee, Henzeh Leeghim

**Affiliations:** 1 MOASOFT Co., Ltd., Seoul 05770, Republic of Korea; yyyang@moasoftware.co.kr (Y.Y.); sskim@moasoftware.co.kr (S.K.); khlee@moasoftware.co.kr (K.L.); 2 Department of Aerospace Engineering, Chosun University, Gwangju 61452, Republic of Korea

**Keywords:** Control Moment Gyro, Disturbance Robust Drive Law, Fast Terminal Sliding Mode Control, UAV attitude stabilization, singularity avoidance

## Abstract

This paper presents a novel control framework for enhancing the attitude stabilization of multirotor UAVs using Control Moment Gyros (CMGs) and a Disturbance Robust Drive Law (DRDL). Due to their lightweight and compact structure, multirotor UAVs are highly susceptible to disturbances such as wind, making it challenging to achieve stable attitude control using rotor thrust alone. To address this issue, we employ CMGs to provide robust attitude control and apply Fast Terminal Sliding Mode Control (FTSMC) to ensure fast and accurate convergence within a finite time. The combination of CMGs’ torque amplification capability with the DRDL enables the system to effectively avoid singularities and maintain stable control performance in the presence of disturbances. Simulation results demonstrate that the CMG-equipped hexarotor utilizing the DRDL rapidly converges to the target attitude despite external disturbances, while minimizing oscillations in both motor speed and gimbal movement. Additionally, compared to the pseudo-inverse control method, the proposed approach significantly improves singularity avoidance and disturbance mitigation. The proposed control framework enhances the stability and reliability of UAV operations and demonstrates its potential for high-performance control in challenging disturbance environments.

## 1. Introduction

Unmanned Aerial Vehicles (UAVs) are generally classified into fixed-wing and rotary-wing types, with multirotors, a subset of rotary-wing UAVs, being actively researched in recent years. Multi-rotors are capable of vertical take-off and landing (VTOL) and hovering in place by controlling the thrust of each rotor, making them suitable for military missions such as surveillance, reconnaissance, target recovery, and damage assessment [1,2,3]. Additionally, their use has expanded into civilian sectors, including aerial photography, environmental monitoring, building inspection, and disaster management [4,5]. However, as the use of multirotors increases, the number of accidents is also rising, with in-flight crashes being the most common [6,7].

One of the main causes of drone accidents is environmental factors such as gusts, which account for approximately 30% of all accidents [8]. Particularly in critical flight stages like landing, these factors significantly affect the stability of drones. This study aims to develop technology that improves the attitude stability of drones in turbulent conditions, such as gusts, to address this vulnerability and enhance operational safety.

Although commercial multirotors are designed to ensure flight stability in wind speeds up to 10 m/s, slight changes in wind speed during flight can still destabilize the aircraft. As a lightweight vehicle, the multirotor has limitations in maintaining flight stability solely through speed control when faced with disturbances like wind gusts. Additionally, the mathematical modeling of multirotor systems can be inaccurate, leading to control performance degradation due to model uncertainty. Therefore, robust control techniques are essential to ensure safe and stable operation in disturbed environments [9,10,11].

Nonlinear control methods used to ensure flight stability include sliding mode control (SMC), backstepping control, neural network control, and adaptive control. While backstepping control offers a systematic approach for nonlinear systems, it requires accurate knowledge of system nonlinearity and can cause rapid output changes [12]. Neural network control optimizes performance by adjusting model variables and weights, but the complexity of the neural network increases the computational burden and slows down control [13]. Adaptive control, whether model-based or non-model-based, also faces challenges in handling complex nonlinear models due to high computational demands.

These existing control techniques have limitations in addressing nonlinear dynamics and managing various environmental factors. Accurate and fast responses are often difficult to achieve in the presence of disturbances and model uncertainty. Sliding Mode Control (SMC) is robust against disturbances and uncertainties, allowing it to handle nonlinear dynamics without linearization by defining a sliding surface using system state variables [14]. However, SMC has two significant drawbacks: first, chattering, which causes high-frequency oscillations, and second, slow convergence of state errors to zero [15,16]. To mitigate these issues, an improved reaching law has been proposed to reduce chattering, and this study applies Fast Terminal Sliding Mode Control (FTSMC) to ensure finite-time convergence [17,18,19,20].

Nevertheless, maintaining attitude stability solely through rotor speed control is challenging under disturbances. To address this, Control Moment Gyros (CMGs), which generate large torques using gyroscopic effects, have been introduced. CMGs are widely used for attitude control in high-agility spacecraft, and recently, they have been applied to multirotor systems to improve flight stability [21,22,23,24,25]. Structurally, CMGs consist of a flywheel and a gimbal and are classified into Variable Speed CMGs (VSCMGs), which control both wheel speed and gimbal angle, and Constant Speed CMGs (CSCMGs), which only adjust the gimbal angle [26,27]. Despite their high performance, CMGs face a geometric singularity problem where they cannot generate control torque in certain directions. This issue arises due to the alignment of the CMG’s gimbal and mounting structure, with pyramid array configurations making it particularly difficult to avoid singularities [28,29,30,31].

In this study, two CSCMGs are mounted side by side along the X-axis of the multirotor to control roll and pitch movements. Yaw control was excluded since the multirotor’s moment of inertia along the Z-axis is relatively large, making it less sensitive to disturbances. However, achieving uniform angular momentum distribution across all axes is difficult, and the maximum angular momentum allowed on each axis is closely related to the initial gimbal angle. If the multirotor fails to restore the initial gimbal angle during hovering, singularities may arise. To address this, we propose an optimal angular momentum vector recovery law to prevent singularities and introduce a Disturbance Robust Drive Law (DRDL) to manage torque adjustments under disturbances [21,32,33,34,35,36].

This study proposes a novel approach to UAV attitude control based on a hexarotor. The research employs Fast Terminal Sliding Mode Control (FTSMC) to ensure robust performance and finite-time convergence under conditions of disturbance and model uncertainty, with stability verified through Lyapunov theory. Additionally, the Disturbance Robust Drive Law (DRDL) is utilized to address the singularity issues of Control Moment Gyros (CMGs), and numerical simulations compare the attitude control performance of the hexarotor with and without CMGs.

The primary contribution of this study goes beyond simply comparing the proposed method with existing control approaches. Instead, it introduces an innovative and practical solution for maintaining stability in disturbed environments by integrating a new actuator, the CMG, into a traditional UAV model. Specifically, the proposed Steering Logic restores the gimbal angles of the CMG to their initial configuration, effectively avoiding singularity issues and ensuring high operability during extended CMG operation. This novel framework addresses critical gaps in previous research, offering a reliable and practical system for UAV attitude control.

## 2. Modeling of UAVs

Multirotors have the advantage of not being constrained by space, as they can perform vertical take-offs and landings (VTOLs) and hover in place through the thrust control of each rotor. Generally, quadrotors are the most commonly used type due to their relatively simple and intuitive operation, but they have the disadvantage of lower flight stability. In this study, a hexarotor, which provides higher output and greater flight stability, and offers a system resistant to disturbances, was used.

**Figure 1:**
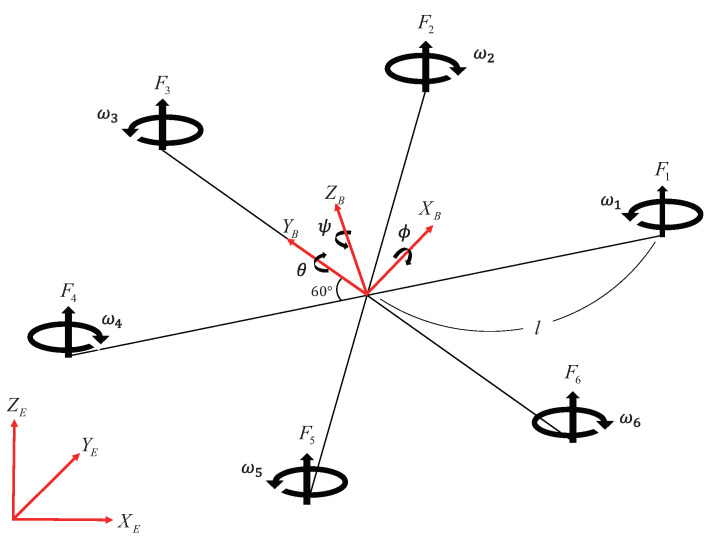
Hexarotor configuration.

To describe the dynamic motion of a hexarotor, it is essential to establish reference frames for both the inertial coordinate system and the body coordinate system. The body coordinate system of the hexarotor is fixed to the observer or the aircraft, while the inertial coordinate system is based on Newton’s first law, which describes a body at rest or in uniform motion unless acted upon by an external force. The inertial coordinate system is typically defined with respect to the center of the Earth. The hexarotor’s structure generates thrust, angular velocity, and torque through its six rotors, as illustrated in Figure 1 [37,38,39]. The dynamic motion of the hexarotor can be characterized by six degrees of freedom and represented by twelve state variables. The components in the inertial frame are defined as position $\zeta ={\left[X_{E},Y_{E},Z_{E}\right]}^{T}$, Euler angles $\eta ={\left[\phi ,\theta ,\psi \right]}^{T}$, and quaternions $Q={\left[q_{1},q_{2},q_{3},q_{4}\right]}^{T}={\left[q,q_{4}\right]}^{T}$. While Euler angles offer intuitive interpretation, there is a risk of gimbal lock, leading to a loss of degrees of freedom [40]. To address this issue and simplify computations, quaternions are employed to handle singularities. In this study, quaternions were used for mathematical modeling, and to facilitate intuitive interpretation of hexarotor operations, they were converted into Euler angles for analysis. In the body frame, velocity is defined as $v={\left[v_{x},v_{y},v_{z}\right]}^{T}$, and angular velocity as $\omega =[\omega_{x},\omega_{y},\omega_{z}]$ [41].

### 2.1. Rotation Matrix

To derive the equation of motion for the hexarotor, it is essential to define the motion in a unified coordinate system. Quaternions are applied based on Euler’s rotation theorem, which allows the representation of rotational motion similarly to the translational motion of any rigid body with a fixed point. Quaternions, also known as Euler parameters, serve as the primary rotational factors and are expressed as four-dimensional vectors, providing an effective and singularity-free method for representing rotational dynamics. Quaternions simplify mathematical modeling and are particularly useful in avoiding singularity issues.

(1) $$\begin{array}{c} q_{1}=p_{1}\sin\left(\alpha /2\right)\\ q_{2}=p_{2}\sin\left(\alpha /2\right)\\ q_{3}=p_{3}\sin\left(\alpha /2\right)\\ q_{0}=\cos\left(\alpha /2\right) \end{array}$$

where α represents the angle of rotation with respect to the unit vector $p=[p_{1},p_{2},p_{3}]$.

Quaternions consist of four elements, and they are subject to the constraint that only one condition must be satisfied.

(2) $$q_{1}^{2}+q_{2}^{2}+q_{3}^{2}+q_{0}^{2}=1$$

The direction cosine matrix from the body frame to the inertial frame is as follows.

(3) $$\begin{array}{c} R=\left(q_{0}^{2}-q^{\prime }q\right)I_{3\times 3}+2\left({\mathrm{qq}}^{\prime }\right)+2q_{0}q^{\times }\\ \;\;\;\;\;=\left[\begin{array}{ccc} q_{0}^{2}+q_{1}^{2}-q_{2}^{2}-q_{3}^{2} & 2\left(q_{1}q_{2}-q_{3}q_{0}\right) & 2\left(q_{2}q_{0}+q_{1}q_{3}\right)\\ 2\left(q_{1}q_{2}+q_{3}q_{0}\right) & q_{0}^{2}-q_{1}^{2}+q_{2}^{2}-q_{3}^{2} & 2\left(q_{2}q_{3}-q_{1}q_{0}\right)\\ 2\left(q_{1}q_{3}-q_{2}q_{0}\right) & 2\left(q_{1}q_{0}+q_{2}q_{3}\right) & q_{0}^{2}-q_{1}^{2}-q_{2}^{2}+q_{3}^{2} \end{array}\right] \end{array}$$

where R is an orthogonal matrix, so $R^{-1}=R^{T}$, and $q^{\times }$ is the matrix representation of the general vector product, which can be defined as a skew-symmetric matrix as follows.

(4) $$q^{\times }=\left[\begin{array}{ccc} 0 & -q_{3} & q_{2}\\ q_{3} & 0 & -q_{1}\\ -q_{2} & q_{1} & 0 \end{array}\right]$$

### 2.2. Force and Torque

The thrust generated by a rotor is determined by the lift coefficient and the rotor’s angular velocity, while the total thrust of the hexarotor is the sum of the thrusts from all six rotors.

(5) $$F_{i}=k\omega_{i}^{2},\;\;\;\;\left(i=1,2,3,4,5,6\right)$$

(6) $$T=\sum_{i=1}^{6}F_{i}=k\sum_{i=1}^{6}\omega_{i}^{2},\;\;T_{B}=\left[\begin{array}{c} 0\\ 0\\ T \end{array}\right]$$

where *k* is the lift coefficient of the rotor and $\omega_{i}$ is the angular speed of each rotor.

The hexarotor rotates 60 degrees about the $Z_{B}$ axis, as shown in Figure 1. Roll, pitch, and yaw moments can be calculated from the $F_{i}$ and $\tau_{i}$ components of the body frame and geometric structure. In this case, the effect of rotor angular acceleration ${\dot{\omega }}_{i}$ is negligible in the torque equation and is therefore ignored.

(7) $$\tau_{i}=b\omega_{i}^{2}+J_{m}{\dot{\omega }}_{i}$$

(8) $$\begin{array}{c} \tau_{\phi }=kl\left(\omega_{3}^{2}-\omega_{6}^{2}+\frac{1}{2}\left(-\omega_{1}^{2}+\omega_{2}^{2}+\omega_{4}^{2}-\omega_{5}^{2}\right)\right)\\ \tau_{\theta }=kl\frac{\sqrt{3}}{2}\left(-\omega_{1}^{2}-\omega_{2}^{2}+\omega_{4}^{2}+\omega_{5}^{2}\right)\\ \tau_{\psi }=b\left(-\omega_{1}^{2}+\omega_{2}^{2}-\omega_{3}^{2}+\omega_{4}^{2}-\omega_{5}^{2}+\omega_{6}^{2}\right) \end{array}$$

where *l* represents the distance from the center of the hexarotor to each propeller, *b* is the drag coefficient, and $J_{m}$ denotes the moment of inertia of the rotor.

By combining the thrust and torque equations, the control input equation for the hexarotor can be derived.

(9) $$\left[\begin{array}{c} \tau_{\phi }\\ \tau_{\theta }\\ \tau_{\psi }\\ T_{B} \end{array}\right]=\left[\begin{array}{c} u_{1}\\ u_{2}\\ u_{3}\\ u_{4} \end{array}\right]=A_{hexa}\left[\begin{array}{c} \omega_{1}^{2}\\ \omega_{2}^{2}\\ \omega_{3}^{2}\\ \omega_{4}^{2}\\ \omega_{5}^{2}\\ \omega_{6}^{2} \end{array}\right],\;\;\;\;\;\;u=A_{hexa}X_{hexa}$$

where $A_{hexa}$ represents the motor driving matrix of the hexarotor.

(10) $$A_{hexa}=\left[\begin{array}{cccccc} -\frac{kl}{2} & \frac{kl}{2} & kl & \frac{kl}{2} & -\frac{kl}{2} & -kl\\ -\frac{kl\sqrt{3}}{2} & -\frac{kl\sqrt{3}}{2} & 0 & \frac{kl\sqrt{3}}{2} & \frac{kl\sqrt{3}}{2} & 0\\ -b & b & -b & b & -b & b\\ k & k & k & k & k & k \end{array}\right]$$

To enhance the operational performance of the hexarotor, the attitude is controlled through the thrust regulation of six motors, which requires considering the mathematical model of the motors. Hence, the simplified mathematical model of the BLDC motor used in the hexarotor is as follows.

(11) $${\dot{\omega }}_{m}=\frac{K_{m}}{J_{m}R_{m}}\left(-K_{b}\omega_{m}+V\right)$$

where $w_{m}$ represents the motor angular velocity, $J_{m}$ denotes the moment of inertia of the motor, $K_{m}$ is the torque constant, $R_{m}$ represents the motor resistance, $K_{b}$ is the back electromotive force constant, and *V* refers to the applied voltage.

### 2.3. Mathematical Model of the UAV

The equation of motion for the attitude and position of the hexarotor can be described by the Newton–Euler equations. In the body frame, the centrifugal force $\omega^{\times }\left(mv\right)$ and the force due to mass acceleration $m\dot{v}$ are equal to the gravitational force $R^{T}G$ and the thrust $T_{B}$ of the UAV. The acceleration in the body frame can be expressed as follows.

(12) $$m\dot{v}+\omega^{\times }\left(mv\right)=R^{T}G+T_{B}$$

where *m* represents the total mass of the hexarotor, and *g* denotes gravitational acceleration.

The expression for acceleration is as follows

(13) $$\dot{v}=R^{T}\left[\begin{array}{c} 0\\ 0\\ g \end{array}\right]-\omega \times v+\frac{1}{m}\left[\begin{array}{c} 0\\ 0\\ T \end{array}\right]$$

To improve the actual flight behavior of the UAV, the aerodynamic effect caused by air resistance must be considered. Therefore, this effect can be mathematically expressed as follows:

(14) $$\dot{v}=R^{T}\left[\begin{array}{c} 0\\ 0\\ g \end{array}\right]-\omega \times v+\frac{1}{m}\left[\begin{array}{c} 0\\ 0\\ T \end{array}\right]-\frac{1}{m}\left[\begin{array}{ccc} A_{x} & 0 & 0\\ 0 & A_{y} & 0\\ 0 & 0 & A_{z} \end{array}\right]\left[\begin{array}{c} v_{x}\\ v_{y}\\ v_{z} \end{array}\right]$$

In the body frame, the sum of the centripetal force $\omega^{\times }J_{hexa}\omega$ and the inertial angular acceleration $J_{hexa}\dot{\omega }$ is equal to the torque $u_{\eta }$. The application of the Newton–Euler equation to rotational motion is as follows.

(15) $$J_{hexa}\dot{\omega }=-\omega^{\times }J_{hexa}\omega +u_{\eta }$$

Adding disturbances here is as follows.

(16) $$\dot{\omega }=J_{hexa}^{-1}\left(-\omega^{\times }J_{hexa}\omega +u_{\eta }+d\right)$$

where

(17) $$J_{hexa}=diag\left(\begin{array}{ccc} J_{hexa,xx} & J_{hexa,yy} & J_{hexa,zz} \end{array}\right)$$

(18) $$d={\left[\begin{array}{ccc} \tau_{\phi ,d} & \tau_{\theta ,d} & \tau_{\psi ,d} \end{array}\right]}^{T}$$

(19) $$u_{\eta }={\left[\begin{array}{ccc} \tau_{\phi } & \tau_{\theta } & \tau_{\psi } \end{array}\right]}^{T}$$

$J_{hexa,xx},J_{hexa,yy}$, and $J_{hexa,zz}$ represent the moments of inertia of the hexarotor along the X-, Y-, and Z-axes, respectively, where d denotes the torque disturbance, and $u_{\eta }$ refers to the torque of the hexarotor.

## 3. Modeling of UAV Using CMGs

The structure of the CMG consists of a gimbal rotor, spin rotor, and flywheel, and it can be categorized into two main types based on the control of the flywheel’s speed. The first is the Variable Speed CMG (VSCMG), which controls both the wheel’s rotational speed and the gimbal angle, and the second is the Constant Speed CMG (CSCMG), which maintains a constant rotational speed while only adjusting the gimbal angle. Additionally, CMGs can be classified according to the number of gimbal axes: single-axis gimbal CMGs and double-axis gimbal CMGs. In this study, two CSCMGs were used for attitude control.

The system is defined by a gimbal frame with unit vectors (a,b,c) and the body frame. Here, *a* epresents the unit vector along the gimbal axis, *b* the unit vector along the spinning axis of the wheel, and *c* the unit vector along the torque axis. The components of the gimbal frame unit vectors are assumed to align with the hexarotor’s body reference frame.

**Figure 2:**
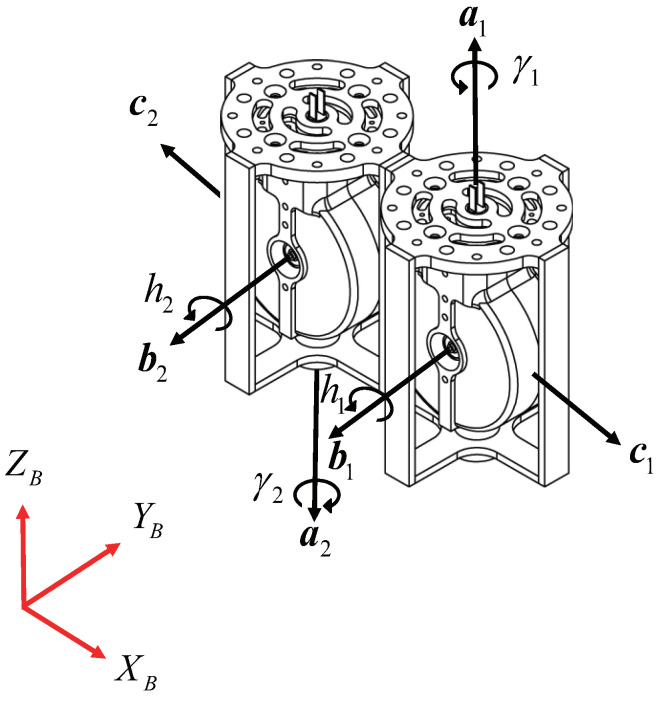
Two CSCMGs configuration.

**Figure 3:**
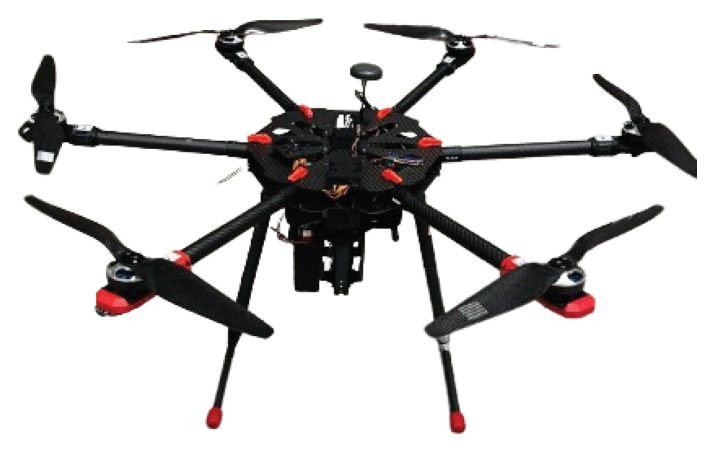
Hexarotor equipped with CSCMGs.

In this study, as illustrated in Figure 2, the CSCMG method was applied to simplify the resolution of singularity problems. Specifically, as shown in Figure 3, two CMGs were mounted side by side along the X-axis of the body frame. This configuration contributes to roll and pitch motion control but does not affect yaw motion. The rationale for this choice is that the moment of inertia along the Z-axis of the hexarotor is larger than that of the other axes, making it less sensitive to disturbances. Therefore, a configuration that only influences roll and pitch motions was selected.

The CMG attached to the hexarotor can be represented in both the body frame of the hexarotor and the gimbal frame of the CMG. The rotation matrix that converts the gimbal frame to the body frame is defined as C.

(20) $$C=\left[\begin{array}{ccc} a & b & c \end{array}\right]$$

The derivatives of the unit vectors derived from the CSCMG are given by the following expression.

(21) $$\begin{array}{c} {\dot{a}}_{i}=0\\ {\dot{b}}_{i}={\dot{\gamma }}_{i}c_{i}\\ {\dot{c}}_{i}=-{\dot{\gamma }}_{i}b_{i} \end{array}$$

In a hexarotor equipped with two CSCMGs, the system rotates with the angular velocity ω of the hexarotor body, the spin rate $\omega_{w}$ of the wheel disk relative to the gimbal frame, and the gimbal rate $\dot{\gamma }$ of the gimbal frame relative to the body frame.

(22) $$\begin{array}{c} {\dot{\gamma }}_{g}={\left[\begin{array}{ccc} \dot{\gamma } & 0 & 0 \end{array}\right]}^{T}\\ {\dot{\omega }}_{w}={\left[\begin{array}{ccc} 0 & \omega_{w} & 0 \end{array}\right]}^{T} \end{array}$$

The angular velocity of the hexarotor relative to the body frame can be converted to the gimbal frame using the following equation.

(23) $$\begin{array}{c} \omega =\omega_{x}x+\omega_{y}y+\omega_{z}z\\ \;\;\;\;\;=\omega_{a}a+\omega_{b}b+\omega_{c}c=C\omega_{g} \end{array}$$

where $\omega_{g}$ is the angular vector with respect to the gimbal frame. The projection of the angular vector onto the gimbal frame follows the relationship shown below.

(24) $$\omega =C\omega_{g}$$

The gimbal direction unit vector a represents an axis fixed to the body frame, and when the gimbal angle γ rotates around this axis, the gimbal angular velocity emerges. The gimbal angular velocity vector is defined as follows.

(25) $${\dot{\gamma }}_{g}=\dot{\gamma }a={\left[\begin{array}{ccc} \dot{\gamma } & 0 & 0 \end{array}\right]}^{T}$$

The total angular momentum of the CSCMG can be represented as the sum of the spin angular momentum and the gimbal angular momentum.

(26) $$h_{cmg}=\sum_{i=1}^{2}h_{i}b_{i}$$

where $h_{cmg}$ represents the angular momentum vector of the CSCMG, $h_{i}=J_{i}\omega_{\omega ,i}$ denotes the internal momentum generated by the *i*-th CSCMG, and $J_{i}$ represents the moment of inertia of the *i*-th CSCMG wheel.

The time derivative of $h_{cmg}$ is equivalent to the torque generated by the CSCMG, as expressed below.

(27) $$\begin{array}{c} {\dot{h}}_{cmg}=\sum_{i=1}^{2}{\dot{h}}_{i}b_{i}+\sum_{i=1}^{2}h_{i}{\dot{b}}_{i}\\ \;\;\;\;\;\;\;\;\;=\sum_{i=1}^{2}J_{i}{\dot{\omega }}_{\omega ,i}b_{i}+\sum_{i=1}^{2}h_{i}{\dot{\gamma }}_{i}c_{i}=u_{cmg} \end{array}$$

where $u_{cmg}$ represents the torque output vector generated by the CSCMG. Since CSCMG was selected in this study, the angular velocity $\omega_{\omega }$ is set to 0. Therefore, it can be expressed as follows.

(28) $$\begin{array}{c} \begin{array}{c} u_{cmg}=\sum_{i=1}^{2}h_{i}{\dot{\gamma }}_{i}c_{i}\\ \;\;\;\;\;\;\;\;\;=\left[\begin{array}{cc} h_{1}c_{1} & h_{2}c_{2} \end{array}\right]\left[\begin{array}{c} {\dot{\gamma }}_{1}\\ {\dot{\gamma }}_{2} \end{array}\right]\\ \;\;\;\;\;\;\;\;\;=A\left(\gamma \right)\dot{\gamma } \end{array} \end{array}$$

(29) $$A\left(\gamma \right)=\left[\begin{array}{cc} -h_{1}\cos\gamma_{1} & h_{2}\cos\gamma_{2}\\ -h_{1}\sin\gamma_{1} & -h_{2}\sin\gamma_{2}\\ 0 & 0 \end{array}\right]$$

where A represents the Jacobian matrix. The detailed derivation of the equations of motion without simplifications is thoroughly described in refs. [27,34,42].

To derive the equation of motion for a hexarotor equipped with two CSCMGs, the total angular momentum vector h of the hexarotor, including the CMGs, is expressed as follows.

(30) $$h=h_{hexa}+h_{cmg}$$

where $h_{hexa}$ represents the angular momentum vector of the hexarotor, and $h_{cmg}$ refers to the angular momentum vector generated by the two CSCMGs.

The equations of motion for the system are derived using Euler’s equation.

(31) $$\dot{h}=u$$

To derive the time variation of the total angular momentum vector h of the hexarotor, the components of Equation (30) are differentiated with respect to time and summarized as follows.

(32) $$J\dot{\omega }=-\omega^{\times }J\omega +\omega^{\times }h_{cmg}-A\left(\gamma \right)\dot{\gamma }+u_{\eta }+d$$

where J is the total moment of inertia of the hexarotor including the two CSCMGs, ω is the angular velocity vector of the hexarotor, $\omega^{\times }$ is the skew-symmetric matrix for the angular velocity vector, and d represents the disturbance.

and

(33) $$\begin{array}{c} J=diag\left(\begin{array}{ccc} J_{xx} & J_{yy} & J_{zz} \end{array}\right)\\ \omega ={\left[\begin{array}{ccc} \omega_{x} & \omega_{y} & \omega_{z} \end{array}\right]}^{T}\\ \omega^{\times }=\left[\begin{array}{ccc} 0 & -\omega_{3} & \omega_{2}\\ \omega_{3} & 0 & -\omega_{1}\\ -\omega_{2} & \omega_{1} & 0 \end{array}\right]\\ \dot{\gamma }={\left[\begin{array}{cc} {\dot{\gamma }}_{1} & {\dot{\gamma }}_{2} \end{array}\right]}^{T} \end{array}$$

## 4. Sliding Mode Control

Most control techniques face limitations in modeling nonlinear dynamics and handling various environmental variables, making it challenging to respond accurately and promptly in the presence of disturbances and model uncertainties. Sliding Mode Control (SMC) is a robust control method that accurately and quickly tracks the target state, and is resilient to disturbances and model uncertainties. SMC can handle nonlinear dynamics without linearization, defining a sliding surface using system state variables and adjusting control variables to drive the system as desired [43]. SMC operates in two phases: the first is the reaching phase, where the control variable moves toward the sliding surface; the second is the sliding phase, where the variable on the surface converges toward the target value. Typically, the control variable is defined as the error between the current and target states, with the controller designed to minimize this error to zero.

A common drawback of conventional sliding mode control is that it takes an infinite amount of time for the state error to converge to zero. To address this, Terminal Sliding Mode Control (TSMC) was introduced, and to further accelerate error convergence, Fast Terminal Sliding Mode Control (FTSMC) was proposed. In this study, FTSMC was used for attitude control, and TSMC was applied for altitude control.

### 4.1. Altitude Control

The Terminal Sliding Mode Control (TSMC) technique is a control method that addresses the issue where variables take infinite time to converge to zero. By applying this technique, convergence to the target value can be guaranteed within a finite time. TSMC was applied for altitude control, and the sliding surface and error equation for this are defined as follows.

(34) $$s_{z}={\dot{e}}_{z}+a_{z}{e_{z}}^{r_{z}}$$

(35) $$e_{z}=z-z_{d}.$$

where $a_{z}$ is a positive gain value greater than zero, and $r_{z}$ is a gain value between 0 and 1. $r_{z}$ must satisfy the condition $0<r_{z}<1$ to ensure finite-time convergence. When $r_{z}>0$, the state variable *e* accelerates its convergence through the nonlinear term $e_{z}^{r_{z}}$. Moreover, $r_{z}<1$ prevents *e* from diverging, enabling stable convergence to e=0. Thus, the range of $r_{z}$ is crucial for achieving the desired convergence properties while maintaining continuity and stability in nonlinear systems.

To obtain the convergence time of TSMC, we assume the sliding surface is as follows.

(36) $$s_{z}={\dot{e}}_{z}+a_{z}{e_{z}}^{r_{z}}=0$$

thus,

(37) $${\dot{e}}_{z}=-a_{z}{e_{z}}^{r_{z}}$$

The above equation can be interpreted as a first-order differential equation with respect to time, and integration is performed to obtain the convergence time.

(38) $$\int_{e_{s}}^{e_{f}}e^{-r_{z}}de=-a_{z}\int_{t_{s}}^{t_{f}}dt$$

where $e_{s}$ represents the initial error, $e_{f}$ represents the final error, $t_{s}$ denotes the start time, and $t_{f}$ denotes the final time.

By applying integration by parts and assuming that the final error $e_{f}$ and the initial time $t_{s}$ are zero,

(39) $${\left[\frac{1}{1-r_{z}}e^{1-r_{z}}\right]}_{e_{s}}^{e_{f}}=-a_{z}{\left[t\right]}_{t_{s}}^{t_{f}}$$

(40) $$\frac{1}{1-r_{z}}e^{1-r_{z}}=-a_{z}t_{f}$$

By rearranging the final time, the convergence time of TSMC can be obtained, ensuring that each variable converges within a finite time.

(41) $$t_{f}=\frac{1}{a_{z}\left(1-r_{z}\right)}e_{s}^{1-r_{z}}$$

To obtain the control input for the altitude control of the hexarotor, the sliding surface equation in Equation (34) can be differentiated with respect to time and rearranged for $u_{z}$.

In this study, the power rate reaching law, consisting of the sum of proportional terms for the sliding surface, was selected. This reaching law is widely used in many studies as it not only reduces chattering but also provides excellent control performance.

(42) $${\dot{s}}_{z}=-\lambda_{z1}s_{z}-\lambda_{z2}{\left|s_{z}\right|}^{p_{z}}sign\left(s_{z}\right)$$

where $\lambda_{z,1}$ and $\lambda_{z,2}$ are positive gain values greater than zero, and $p_{z}$ is a gain value between 0 and 1. The gain values were selected within the range that optimizes system performance based on the simulation results.

The sliding surface in Equation (34) has an issue where an imaginary value appears when the state variable e is negative. To address this problem, we use the following modified sliding surface.

(43) $$s_{z}={\dot{e}}_{z}+a_{z}{\left|e_{z}\right|}^{r_{z}}sign\left(e_{z}\right)$$

where sign (·) are signum functions.

Using the modified sliding surface, the control input for the altitude control of the hexarotor can be derived as follows.

(44) $$u_{4}=\frac{m}{R_{z}}\left(-a_{z}r_{z}{\left|e_{z}\right|}^{r_{z}-1}{\dot{e}}_{z}+g+{\dot{s}}_{z}\right)$$

where $R_{z}$ corresponds to the values in the third row and third column of Equation (3).

The Lyapunov function is used to verify the stability of the system with the designed controller. The objective of the controller is to drive the sliding variable to zero, and the Lyapunov candidate function is defined as follows.

(45) $$V=\frac{1}{2}s_{z}^{2}$$

Differentiating the Lyapunov candidate function is not feasible due to the discontinuity at $s_{z}=0$. To address this issue, a sigmoid-based approximation is used, ensuring continuity. The differentiation of the Lyapunov candidate function with the proposed modification is as follows:

(46) $$\dot{V}=s_{z}{\dot{s}}_{z}=s_{z}\left(-\lambda_{z1}s_{z}-\lambda_{z2}{\left|s_{z}\right|}^{p_{z}}\frac{s_{z}}{\sqrt{s_{z}^{2}+\varepsilon }}\right)<0$$

Therefore, as it can be verified that the value is less than 0, the controller is Lyapunov stable, and the sliding surface converges to zero after a sufficient amount of time.

### 4.2. Attitude Control

Fast Terminal Sliding Mode Control (FTSMC) is a control technique that accelerates the convergence speed compared to conventional TSMC. The sliding surface of FTSMC is defined as follows.

(47) $$s=\dot{e}+a_{\eta }e+b_{\eta }{\dot{e}}^{r}$$

where $a_{\eta }$ and $b_{\eta }$ are positive gain values greater than zero, and *r* represents gain values between 0 and 1.

FTSMC ensures a faster convergence rate compared to TSMC. The method of calculating the convergence speed is the same as that of TSMC, guaranteeing that each variable converges to 0 within the time $t=ln\left(\left(a_{\eta }e_{s}^{1-r}+b_{\eta }\right)/b_{\eta }\right)/\left(a_{\eta }\left(1-r\right)\right)$. Similar to the sliding surface in TSMC, the issue of imaginary numbers can arise when the variable is negative. This problem is solved in the same way as TSMC, and for attitude control, the error equation is defined using the desired quaternion $q_{d}$, with the sliding surface where the sliding variable is 0 being as follows.

(48) $$s_{\eta }={\left[s_{\phi },s_{\theta },s_{\psi }\right]}^{T}=\omega +a_{\eta }Q_{e}+b_{\eta }G{\left(Q_{e}\right)}^{r}\mathrm{sgn}\left(Q_{e}\right)$$

(49) $$Q_{e}=\left[\begin{array}{c} q_{e}\\ q_{e,4} \end{array}\right]=\left[\begin{array}{cc} q_{d,4}I_{3\times 3}-q_{d}^{\times } & -q_{d}\\ q_{d}^{T} & q_{d,4} \end{array}\right]\left[\begin{array}{c} q\\ q_{0} \end{array}\right]$$

To obtain the control input for attitude control of the hexarotor, the equation for the sliding variable is differentiated with respect to time as follows.

(50) $${\dot{s}}_{\eta }=-\lambda_{1}s_{\eta }-\lambda_{2}G{\left(s_{\eta }\right)}^{p}\mathrm{sgn}\left(s_{\eta }\right)$$

where sign(·) represents signum functions, $\lambda_{1}$ and $\lambda_{2}$ are positive gain values greater than zero, and *p* is a gain value between 0 and 1.

Using the above equations, the control input for the attitude of the hexarotor can be derived as follows.

(51) $$u_{\eta }=\omega^{\times }J!-J\left(-\dot{s}+\left(a_{\eta }I_{3\times 3}+b_{\eta }rG{\left(Q_{e}\right)}^{r-1}\right)\frac{1}{2}\left(Q_{e}^{\times }+q_{0}I_{3}\right)\omega \right).$$

where

(52) $$G\left(s\right)=diag\left(\left|s_{\phi }\right|,\left|s_{\theta }\right|,\left|s_{\psi }\right|\right)$$

(53) $$\mathrm{sgn}\left(q_{i}\right)={\left[sign\left(q_{1}\right),sign\left(q_{2}\right),sign\left(q_{3}\right)\right]}^{T}$$

Lyapunov theory is applied to verify the stability of the system using the designed controller. Additionally, the robustness of FTSMC is confirmed through the modeling Equation (16), which considers disturbances. The goal of the controller is to reduce the sliding variable to zero, and the Lyapunov candidate function is defined as follows.

(54) $$V=\frac{1}{2}s_{\eta }^{T}Js_{\eta }$$

Differentiating the Lyapunov candidate function yields the following expression.

(55) $$\dot{V}=s_{\eta }^{T}J{\dot{s}}_{\eta }={s_{\eta }}^{T}\left(-\lambda_{1}s_{\eta }-\lambda_{2}G{\left(s_{\eta }\right)}^{p}sgn\left(s_{\eta }\right)\right)<0$$

Therefore, since it can be observed that the value is less than 0, the controller is Lyapunov stable, and the sliding surface converges to 0 after sufficient time.

## 5. CMG Drive Law

In this study, instead of the pyramidal arrangement commonly used in spacecraft, a CMG with a relatively simple singularity space, as shown in Figure 2, was selected as the actuator for attitude control. To implement the three-axis control torque commands generated for hexarotor attitude control, an actuator command distribution law is required.

### 5.1. Pseudo−Inverse Drive Law

The pseudo-inverse drive law is commonly used for torque distribution. The equation for calculating the gimbal angular velocity vector using the pseudo-inverse drive law with respect to a given control torque command $u_{c}$ is as follows:

(56) $$\dot{\gamma }=A^{T}{\left(AA^{T}\right)}^{-1}u_{c}$$

However, since the Jacobian matrix used in the pseudo-inverse drive law is a function of the gimbal angles, there remains the possibility of encountering singularities at specific gimbal angle configurations. Singularities are classified as internal or external, depending on how the CMG angular momentum vectors are aligned. When two CMGs are aligned in opposite directions, the total angular momentum vector becomes zero, causing an internal singularity. Conversely, when two CMGs are aligned in the same direction, the total angular momentum vector doubles, resulting in an external singularity. Under such alignment conditions, the CMGs cannot generate the desired control torque in the specified direction.

### 5.2. Angular Momentum Vector Recovery Drive Law

The initial gimbal angle position is closely associated with the rotational maneuverability of the hexarotor. The angular momentum vector $h_{cmg}$ for each axis is a function of the gimbal angle $\gamma_{i}$, and its value continuously changes with variations in the CMG gimbal vector. To maintain consistent maneuverability throughout the maneuvering period, the allowable angular momentum and gimbal operating range for each axis must remain unchanged before the start of attitude maneuvers. In other words, upon completion of the maneuver, the gimbal angle should always return to its initially defined direction, which implies that the angular momentum vector must also return to its initial value. Failure to do so may gradually lead to a singularity.

In this study, a gimbal vector recovery drive law was derived using an optimization technique. The performance index to be minimized is defined as follows:

(57) $$J=\frac{1}{2}{\dot{\gamma }}^{T}M\dot{\gamma }+\frac{1}{2}{\left(\dot{\gamma }-{\dot{\gamma }}_{d}\right)}^{T}N\left(\dot{\gamma }-{\dot{\gamma }}_{d}\right)$$

where *M* and *N* are symmetric positive definite weighting matrices. At this point, the constraint equation $u_{c}$ is introduced. The first term of the equation contributes to minimizing the sum of the control torque input squares, similar to the conventional pseudo-inverse method, while the second term aims to minimize the difference between the current gimbal angular velocity of the CSCMG and the desired or pre-set angular velocity by the user.

The desired gimbal angular velocity $\gamma_{d}$ is defined as:

(58) $${\dot{\gamma }}_{d}=\left(\gamma -\gamma_{d}\right)/\Delta t$$

By applying the optimality condition, the following solution is derived for the performance index J [44]:

(59) $$\dot{\gamma }=WA^{T}{\left(\mathrm{AW}A^{T}\right)}^{-1}u_{c}+\left[WA^{T}{\left(\mathrm{AW}A^{T}\right)}^{-1}\mathrm{AW}-W\right]g$$

where $W={\left(M+N\right)}^{-1}$, and g is a non-zero gradient vector defined as $g=N(\gamma -\gamma_{d})/\Delta t$. Accordingly, the second term on the right-hand side corresponds to the null vector for angular momentum recovery, while the first term corresponds to the pseudo-inverse drive law.

When the weighting matrices *M* and *N* are set as $M=\alpha I_{2\times 2}$ and $N=\beta I_{2\times 2}$, the Angular Momentum Vector Recovery Drive Law (AMVRSL) for obtaining the CSCMG angular acceleration vector is simplified as follows:

(60) $$\dot{\gamma }=A^{†}u_{c}+n$$

(61) $$A^{†}=A^{T}{\left(AA^{T}\right)}^{-1}$$

(62) $$n=\left[A^{†}A-I_{2\times 2}\right]\&\left(\gamma -\gamma_{d}\right)$$

where n is null vector, and α and β are positive constants. AMVRSL is activated during the final maneuver section. The factor ς is defined using the S-shaped sigmoid function as follows:

(63) $$\varsigma =\frac{\beta }{\left[(\alpha +\beta )\Delta t\right]}=\frac{L}{1+e^{-k\sigma }}$$

where *K* and *L* are constants, $∥e∥$ is the norm of the attitude error, and $∥\omega ∥$ is the angular velocity vector of the hexarotor. The variable σ is defined as:

(64) $$\sigma =\frac{1}{∥e∥+k_{0}∥\omega ∥}-\varepsilon$$

This means that both the attitude and angular velocity converge to small values during the final maneuvering phase. Thus, ς is designed to ensure that AMVRSL is only activated in the last phase of the maneuver.

In this study, the angular momentum recovery strategy is activated or deactivated based on the condition $∥e∥+∥\omega ∥<\upsilon$. When activated, η is set to 1/υ=100. Additionally, the maximum value of the null vector is constrained by the following standard:

(65) $$n=\left\{\begin{array}{cc} \bar{n}\;\frac{n}{{∥n∥}_{\infty }\;}\;\;\;\;, & {∥n∥}_{\infty }\geq \bar{n}\\ n\;\;\;\;\;\;\;\;\;\;\;\;\;\;\;\;, & {∥n∥}_{\infty }<\bar{n} \end{array}\right.$$

where ${∥n∥}_{\infty }$ represents the infinity norm of the vector *n*.

### 5.3. Disturbance Robust Drive Law

During the actual flight of the hexarotor, various factors, such as wind disturbances, can affect the attitude of the hexarotor. These disturbances also interfere with the operation of the CMG, leading to singularity issues within the system. Specifically, external disturbances prevent the gimbal from returning to its original position. As observed in previous case studies, the unwanted external disturbance torques cannot be effectively managed using only the CMG-generated torque. To address this disturbance-induced singularity problem, the hexarotor’s motor torque will now be utilized in conjunction with the CMG.

The dynamic equation of the hexarotor with a CMG can be expressed as follows:

(66) $$\begin{array}{c} J\dot{\omega }+\omega^{\times }J\omega +\omega^{\times }h_{cmg}=-A_{hexa}\omega^{2}-A\left(\gamma \right)\dot{\gamma }+d\\ \;\;\;\;\;\;\;\;\;\;\;\;\;\;\;\;\;\;\;\;\;\;\;\;\;\;\;\;\;\;\;\;\;\;\;\;\;\;\;\;\;\;\;\;\;\;=-Bu+d \end{array}$$

where $u={\left[\begin{array}{cc} \omega_{i}^{2} & \dot{\gamma } \end{array}\right]}^{T}\in R^{8}$ represents the control input, and $B=\left[\begin{array}{cc} A_{\mathrm{hexa}} & A_{\mathrm{cmg}} \end{array}\right]\in R^{4\times 8}$.

Through the motion equation of the hexarotor using a CMG, it is corrected with a drive law robust against disturbances. The optimization problem to minimize the performance index is defined as follows:

(67) $$J=\frac{1}{2}u^{T}Pu+\frac{1}{2}{\left(u-u_{d}\right)}^{T}H\left(u-u_{d}\right)$$

subject to the constraint:

(68) $$u_{c}=Bu$$

where $P\in R^{8\times 8}$ and $H\in R^{8\times 8}$ are symmetric positive definite weighting matrices, and $ud={\left[\begin{array}{cc} \omega {i,d}^{2} & {\dot{\gamma }}_{d} \end{array}\right]}^{T}\in R^{8}$ represents the desired control vector.

By applying the optimality condition [44], the minimum solution is derived as:

(69) $$u=WB^{T}{\left(\mathrm{BW}B^{T}\right)}^{-1}u_{c}+\left[WB^{T}{\left(\mathrm{BW}B^{T}\right)}^{-1}\mathrm{BW}-W\right]g$$

where W and g are the gradient vector forcing the gimbal to return to its preferred initial position, P and H are the weighting matrices.

Since there are multiple solutions satisfying the equation, the pseudo-inverse solution for the first term on the right-hand side is preferred. The solution can be rewritten as:

(70) $$u=A^{T}{\left(AA^{T}\right)}^{-1}u_{c}+n$$

(71) $$n=\left[WB^{T}{\left(\mathrm{BW}B^{T}\right)}^{-1}\mathrm{BW}-W\right]\varsigma \left(u-u_{d}\right)$$

where n is the null vector, and

(72) $$\begin{array}{c} P=\left[\begin{array}{cc} p_{hexa}I_{6\times 6} & O_{6\times 2}\\ O_{2\times 6} & p_{cmg}I_{2\times 2} \end{array}\right]\in R^{8\times 8}\\ H=\left[\begin{array}{cc} q_{hexa}I_{6\times 6} & O_{6\times 2}\\ O_{2\times 6} & q_{cmg}I_{2\times 2} \end{array}\right]\in R^{8\times 8}\\ W={\left(P+H\right)}^{-1}\in R^{8\times 8}\\ g=H\left(u-u_{d}\right)\in R^{7} \end{array}$$

where $0_{m\times n}$ represents an m×n zero matrix, and $p_{hexa}$, $p_{cmg}$, $q_{hexa}$, and $q_{cmg}$ are the respective weighting constants.

## 6. Simulation Study

### 6.1. Simulation Configuration

**Figure 4:**
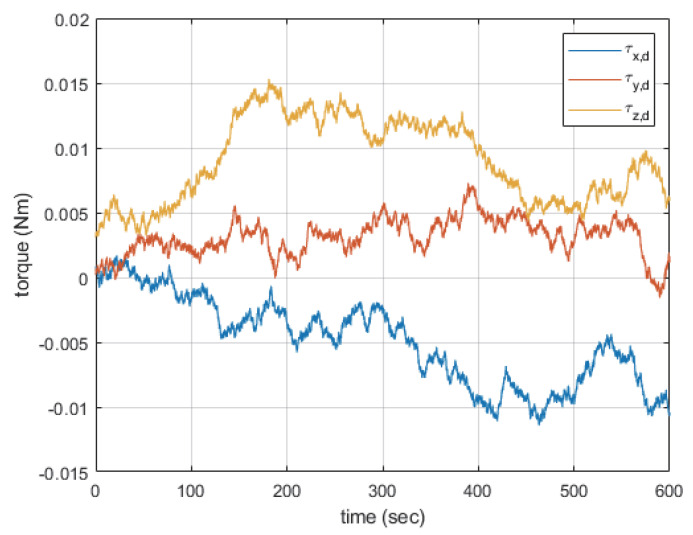
Disturbance scenario.

The numerical simulation related to the drive law was performed. To achieve maximum maneuverability of the hexarotor equipped with two CSCMGs, the simulation was conducted under the conditions outlined in Table 1. The drive law used to derive the angular velocity of the CSCMG gimbal was compared and analyzed using both the pseudo-inverse drive law and the DRDL. The parameters for the DRDL are shown in Table 2. The disturbance scenario was designed by mathematically implementing wind disturbances as random walk disturbance torques, based on the torque values presented in Figure 4. The simulation was performed by incorporating the external torque values into the hexarotor modeling equations.

### 6.2. Pseudo-Inverse Drive Law Simulation Result

**Figure 5:**
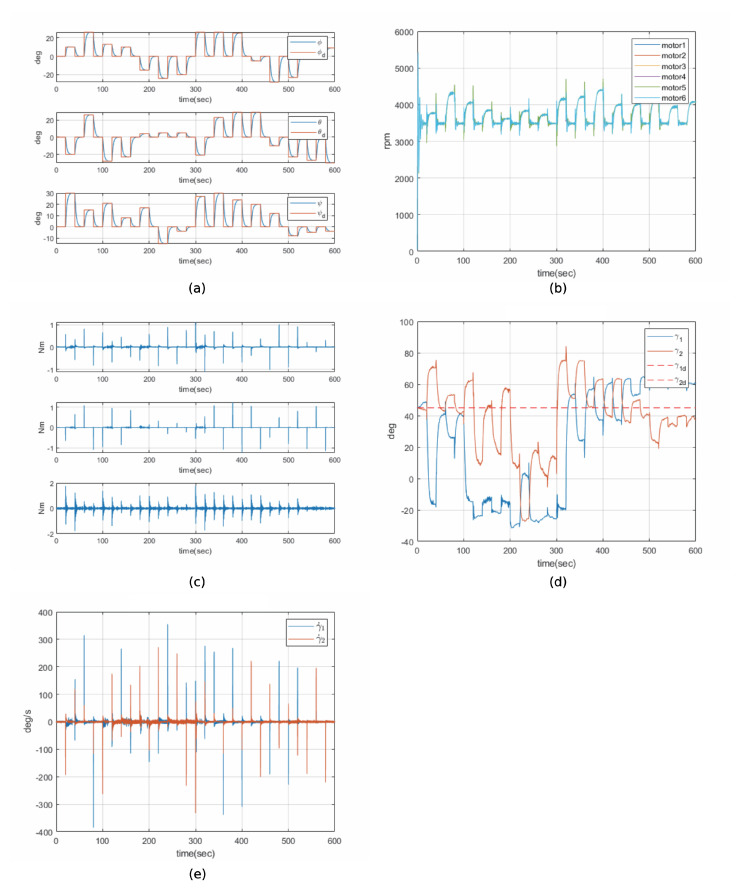
Histories of pseudo−inverse drive law: (**a**) attitude of hexarotor, (**b**) motor speed of hexarotor, (**c**) torque of hexarotor, (**d**) gimbal angle of CMG, (**e**) gimbal rate of CMG.

Simulation results based on the pseudo-inverse drive law demonstrate how the hexarotor system achieves control torque without explicitly avoiding singularities. This method provides a minimum-norm solution but does not guarantee singularity avoidance. Figure 5a–e present state graphs of a hexarotor equipped with CMGs under disturbance conditions, controlled by the pseudo-inverse drive law.

Figure 5a shows the attitude response to various attitude commands. While there is a slight deviation due to the disturbances, it can be observed that all responses converge to the target attitude.

Figure 5b,c illustrate the motor speed and torque of the hexarotor, respectively. During command transitions, oscillations appeared in both the motor speed and torque. These oscillations are known as “chattering”, caused by the sign function included in the reaching law within the controller. The chattering was particularly pronounced during attitude transitions and disturbance handling.

Figure 5d,e depict the gimbal angle and angular velocity of the CMGs. A noticeable drift in the gimbal angle from the initially set 45 degrees was observed due to disturbances. The gimbal was unable to return to its original position, indicating that the pseudo-inverse drive law struggles to handle singularity issues arising from disturbances. Instabilities in the gimbal’s angular velocity were also evident, with significant chattering observed.

In conclusion, under the pseudo-inverse drive law, the hexarotor equipped with CMGs experienced difficulties in maintaining accurate control torque in a disturbance environment, and oscillations caused by disturbances were only limitedly mitigated.

### 6.3. Disturbance Robust Drive Law Simulation Result

**Figure 6:**
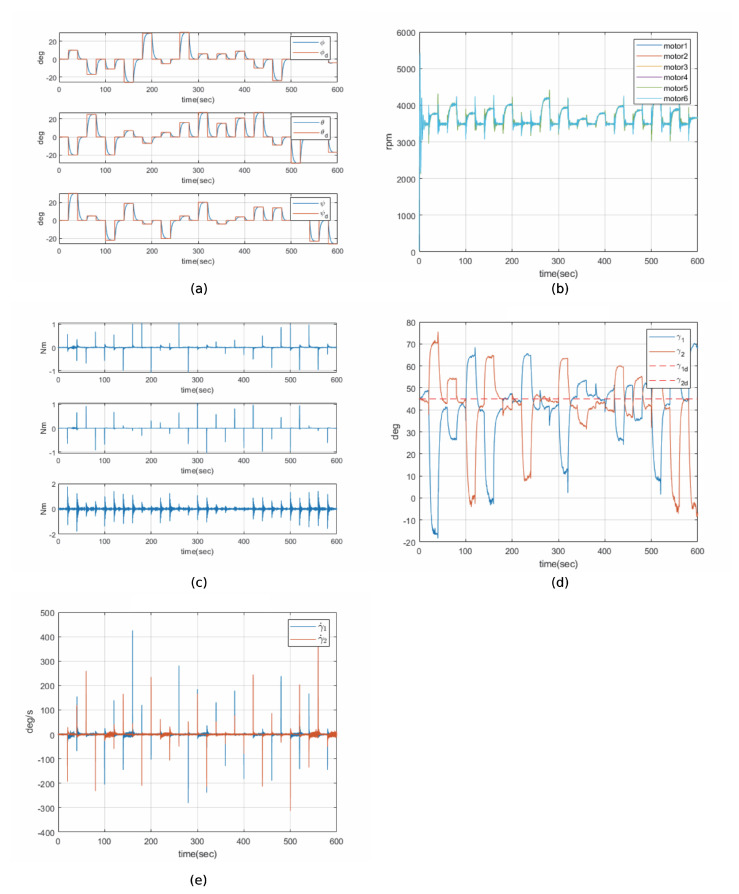
Histories of Disturbance Robust Drive Law: (**a**) attitude of hexarotor, (**b**) motor speed of hexarotor, (**c**) torque of hexarotor, (**d**) gimbal angle of CMG, (**e**) gimbal rate of CMG.

In contrast, the DRDL simulation exhibited enhanced performance in a disturbance environment. This control law is specifically designed to improve the system’s robustness against external disturbances. The simulation results demonstrate its superior ability to effectively avoid singularities. Figure 6a–e depict the state graphs of the hexarotor equipped with CMGs operating under the DRDL control method in a disturbance environment.

Figure 6a shows the attitude response to multiple attitude commands. As in the pseudo-inverse simulation, minor errors are observed due to disturbances; however, all responses converge to the desired attitude.

Figure 6b,c present the motor speed and torque of the hexarotor, respectively. Compared to the pseudo-inverse method, the DRDL significantly reduces chattering in both motor speed and torque. Even during attitude command transitions, the oscillations are less severe, enabling the motors to operate more stably and generate torque more efficiently.

Figure 6d,e illustrate the gimbal angles and angular velocities of the CMGs. The initial gimbal angle was set to 45 degrees, and it was observed that, during multi-attitude command maneuvers, the gimbal angles remained substantially more stable compared to the pseudo-inverse drive law. Furthermore, the angular velocities of the two CMGs were also highly stable within hardware limits. The hexarotor with CMGs controlled by the DRDL was able to avoid singularity issues, even in a disturbance environment.

In conclusion, the DRDL demonstrated superior performance in both singularity avoidance and disturbance management, ensuring stable CMG operation and effective attitude control under disturbed conditions.

## 7. Conclusions

This study proposed an attitude stabilization framework for multirotors using Control Moment Gyros (CMGs) and an enhanced control strategy called the Disturbance Robust Drive Law (DRDL). The focus was on addressing the challenges posed by external disturbances such as wind gusts, which can degrade the flight stability of lightweight UAVs. Through the use of Fast Terminal Sliding Mode Control (FTSMC) for attitude stabilization, we ensured finite-time convergence, guaranteeing improved system response even under the presence of disturbances. This approach, along with the application of the DRDL, aimed at mitigating issues like singularity and ensuring robust control performance.

CMGs demonstrated their ability to generate large torques with minimal input, which allowed for improved stability and control, especially during multi-attitude commands. When combined with the DRDL, the system exhibited superior singularity avoidance and robust behavior in disturbed environments. Unlike the pseudo-inverse drive law, which showed limitations in handling disturbances and singularities, the DRDL method allowed the UAV to maintain its gimbal angles more effectively, avoiding the drift that typically results from external interference. Additionally, the DRDL successfully minimized chattering, ensuring that the motors operated efficiently without excessive oscillations.

In conclusion, this study confirmed that integrating CMGs with the DRDL significantly enhances the robustness and stability of multirotor systems under challenging environmental conditions. The combined approach of using CMGs for torque amplification, FTSMC for rapid response, and the DRDL for singularity avoidance provides a reliable and high-performance solution for UAV attitude control. Future work may further optimize the integration of these methods to improve UAV flight capabilities in more complex and varied disturbance scenarios.

## Figures and Tables

**Table 1:** Simulation configuration conditions.

Parameter	Values	Parameter	Values
Number of commands	30	Total time (sec)	600
One command time (sec)	20	Command maximum (deg)	±30

**Table 2:** Simulation parameters of DRDL.

Parameter	Values	Parameter	Values
*L*	1	$p_{hexa}$	1 × 10^5^
$k_{0}$	1	$q_{hexa}$	1 × 10^5^
*k*	1	$p_{cmg}$	10
ε	500	$q_{cmg}$	1
$\bar{n}$	0.1		

## Data Availability

Data are contained within the article.
